# Supplementary material for: Molecular Dynamics-Assisted Design of High Temperature-Resistant Polyacrylamide/Poloxamer Interpenetrating Network Hydrogels
Source: Molecules. 2022 Aug 21;27(16):5326. doi: 10.3390/molecules27165326 (PMC9414860; doi:10.3390/molecules27165326)
Supplement: Supplementary file 1 [file molecules-27-05326-s001.zip › molecules-1842230-supplementary.pdf]

Supporting Information

Molecular Dynamics-assisted Design of High Temperature-Resistant Polyacrylamide/Poloxamer Interpenetrating Network Hydrogels

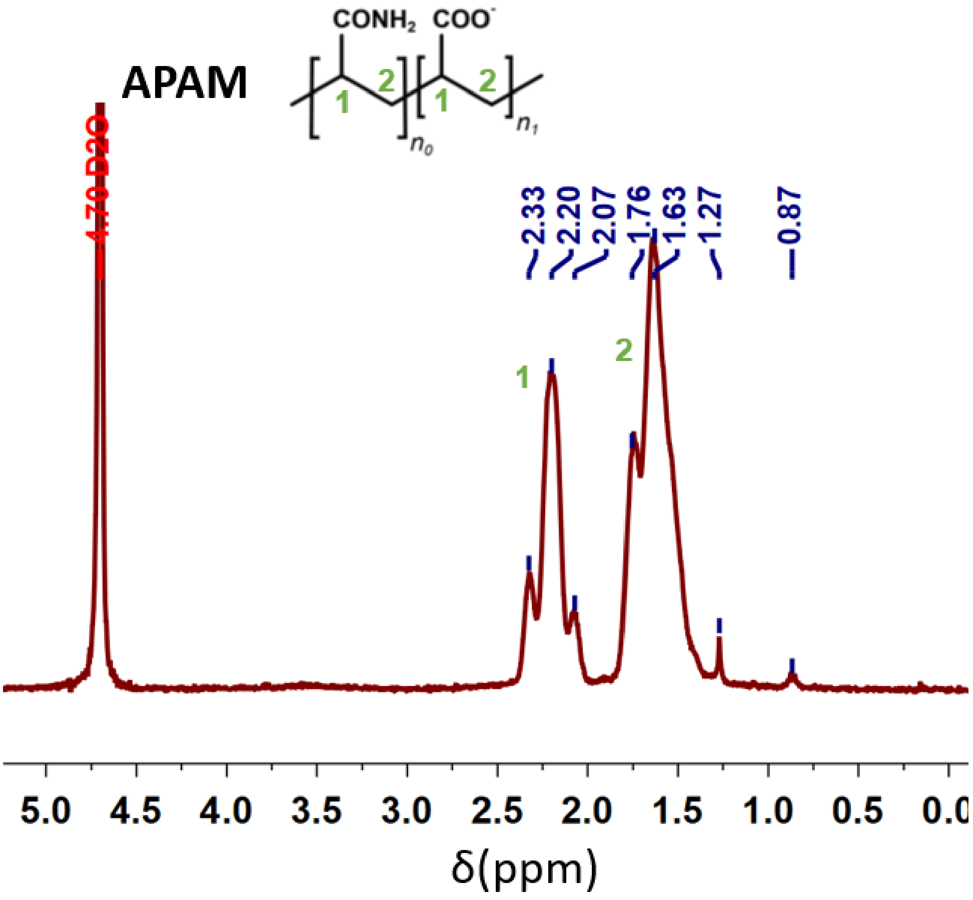

Figure. S1 The full ^{1}H NMR spectrum of the **APAM**. The peak for hydrogens on methylene groups(-CH-) attached to carboxyl or amide groups is observed around δ 2.20. The hydrogen of -CH_2 on the polymer chain appears around δ1.63.

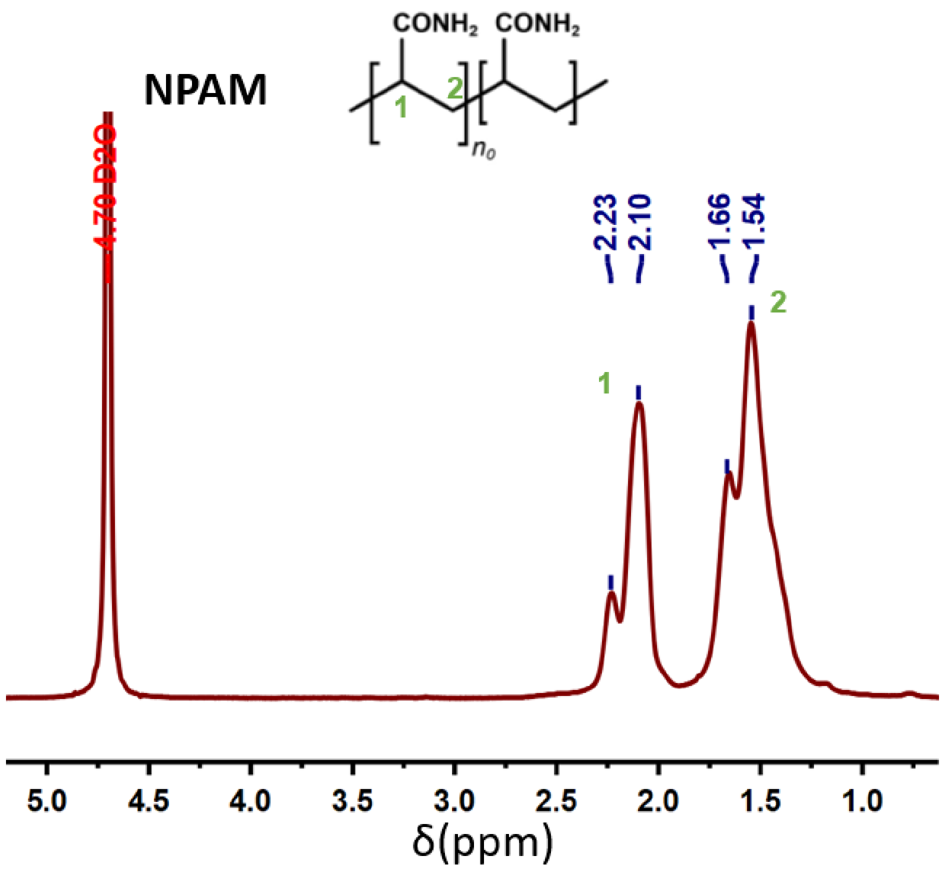

Figure. S2 The full ^{1}H NMR spectrum of the **NPAM**. The peak of the hydrogen on the methylene (-CH-) attached to the carboxyl or amide group is around δ 2.10. The hydrogen of -CH_2 on the polymer chain appears around δ1.66.

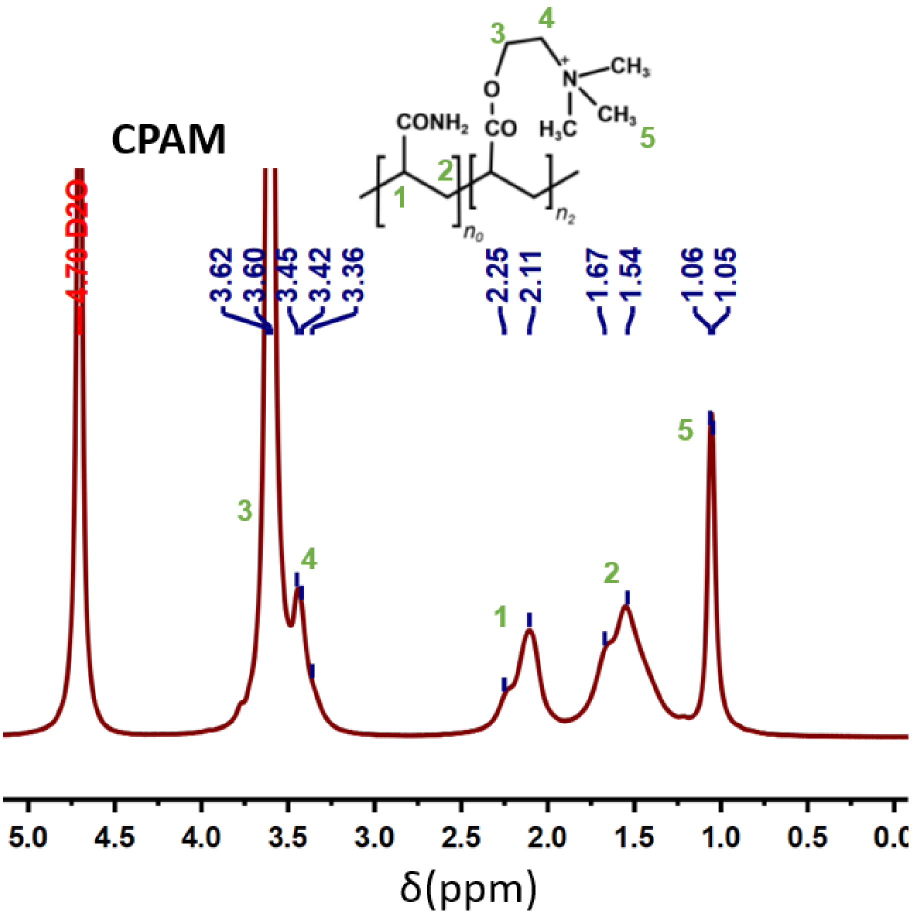

Figure. S3 The full ^{1}H NMR spectrum of the **CPAM**. The peak for hydrogens on methylene groups(-CH-) attached to carboxyl or amide groups is observed around δ 2.11. The hydrogen of -CH_2 on the polymer chain appears around δ1.54. In addition, the hydrogens of methyl groups (-CH_3) located on branched chains are observed around δ 1.05. The hydrogens on the methylene groups on the branch are located at δ 3.30 and δ 3.60, respectively.

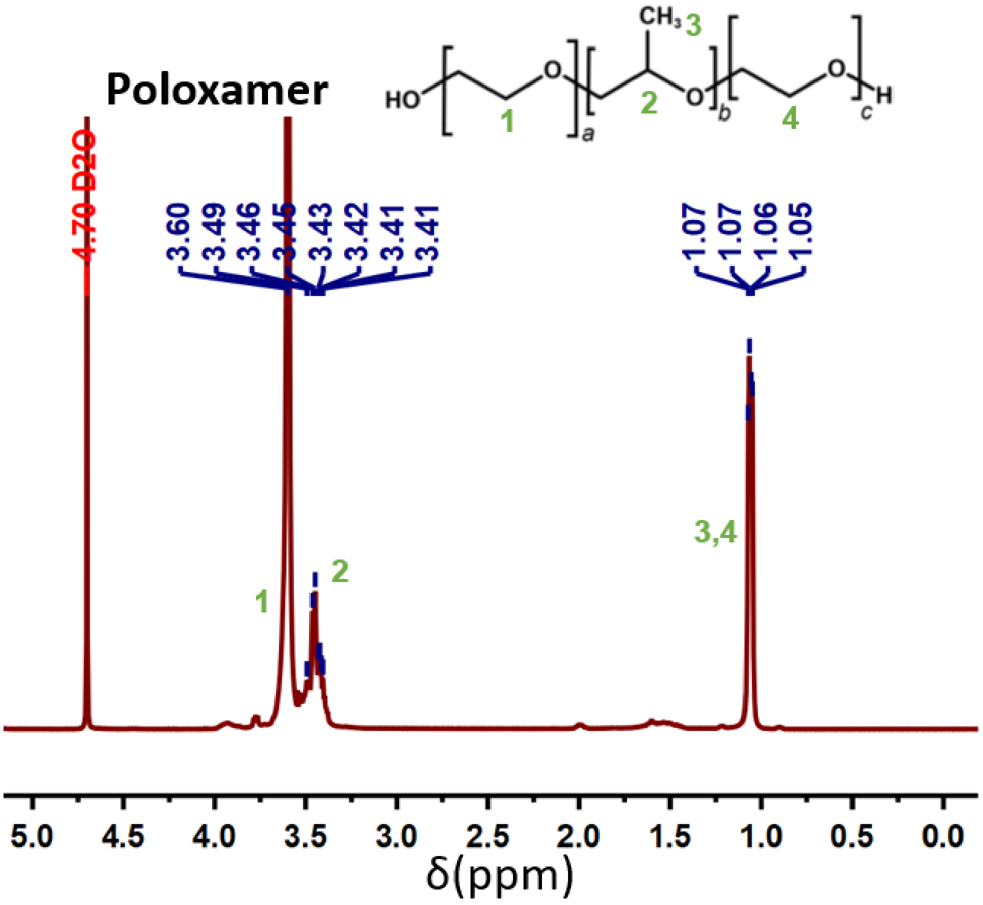

Figure. S4 The full ^{1}H NMR spectrum of the poloxamer. The hydrogen of the methyl group (-CH_3) on the molecular chain is located at δ 2.20. In addition, the hydrogen peak on the methylene group (-CH-) attached to the methyl group is around δ 3.41. The peak appearing around δ 3.60 is the hydrogen on the methylene (-O-CH-) attached to the ether group.

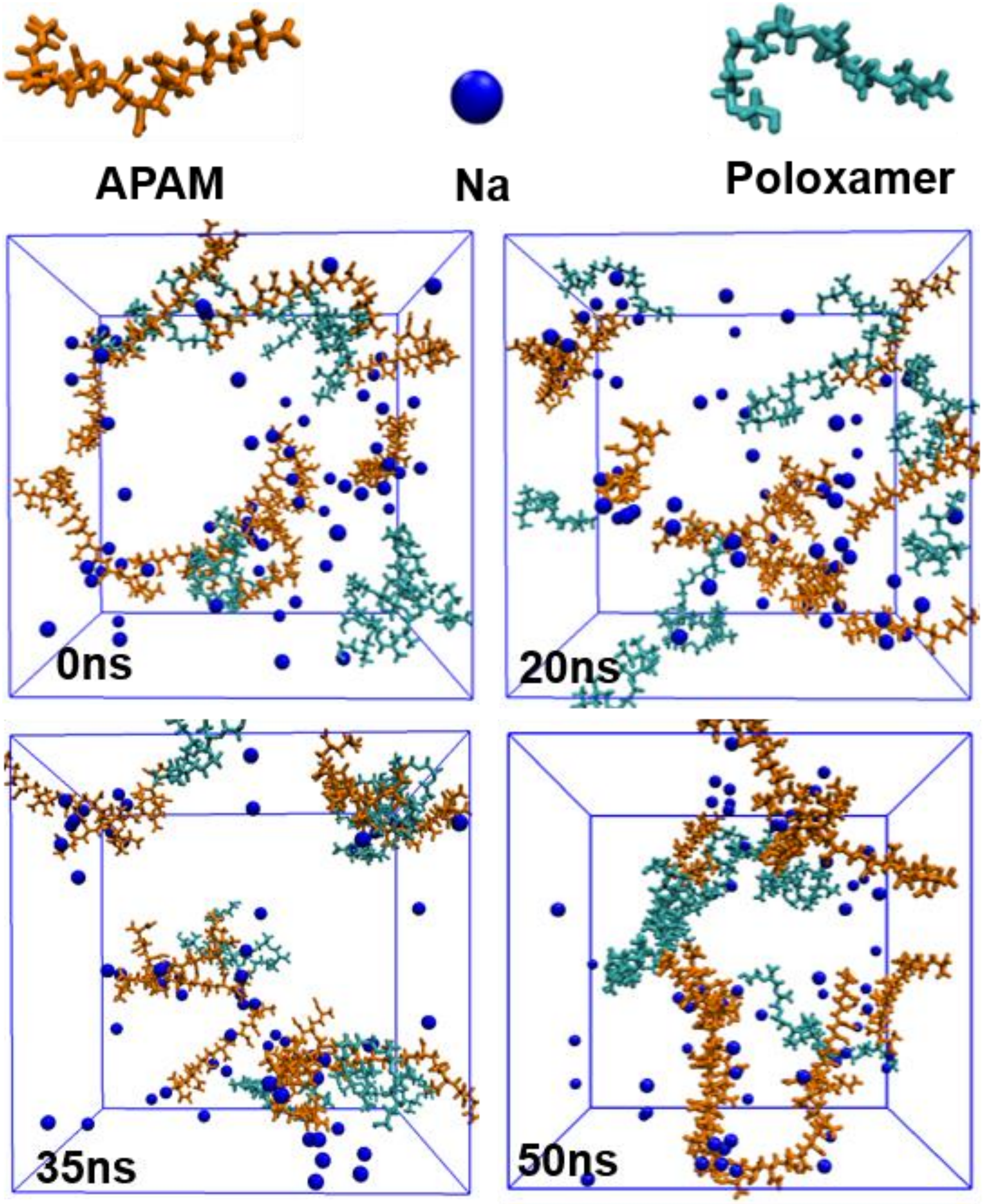

Figure. S5 Molecular dynamics process of the interaction between **APAM** and poloxamer.

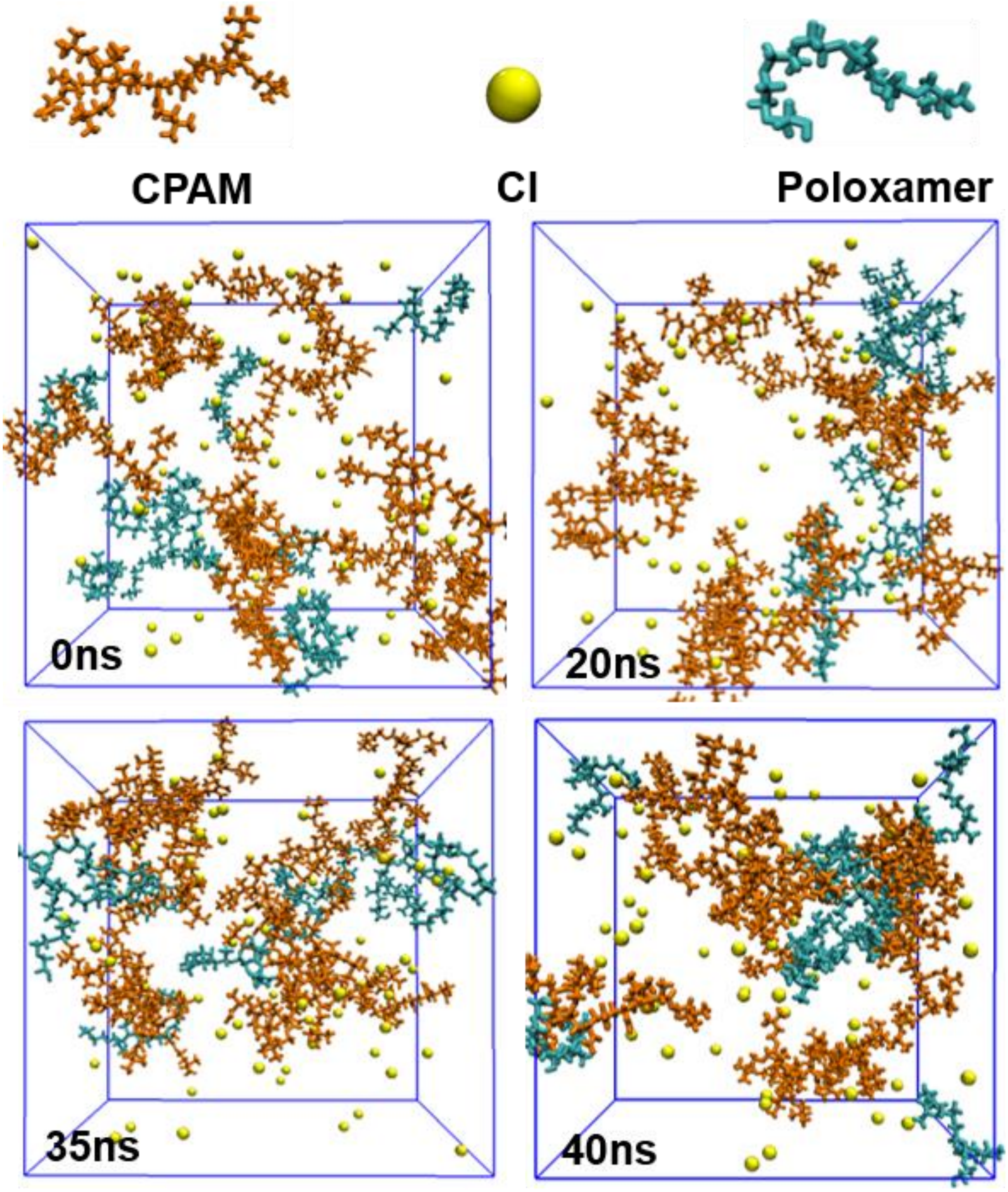

Figure. S6 Molecular dynamics process of the interaction between **CPAM** and poloxamer.

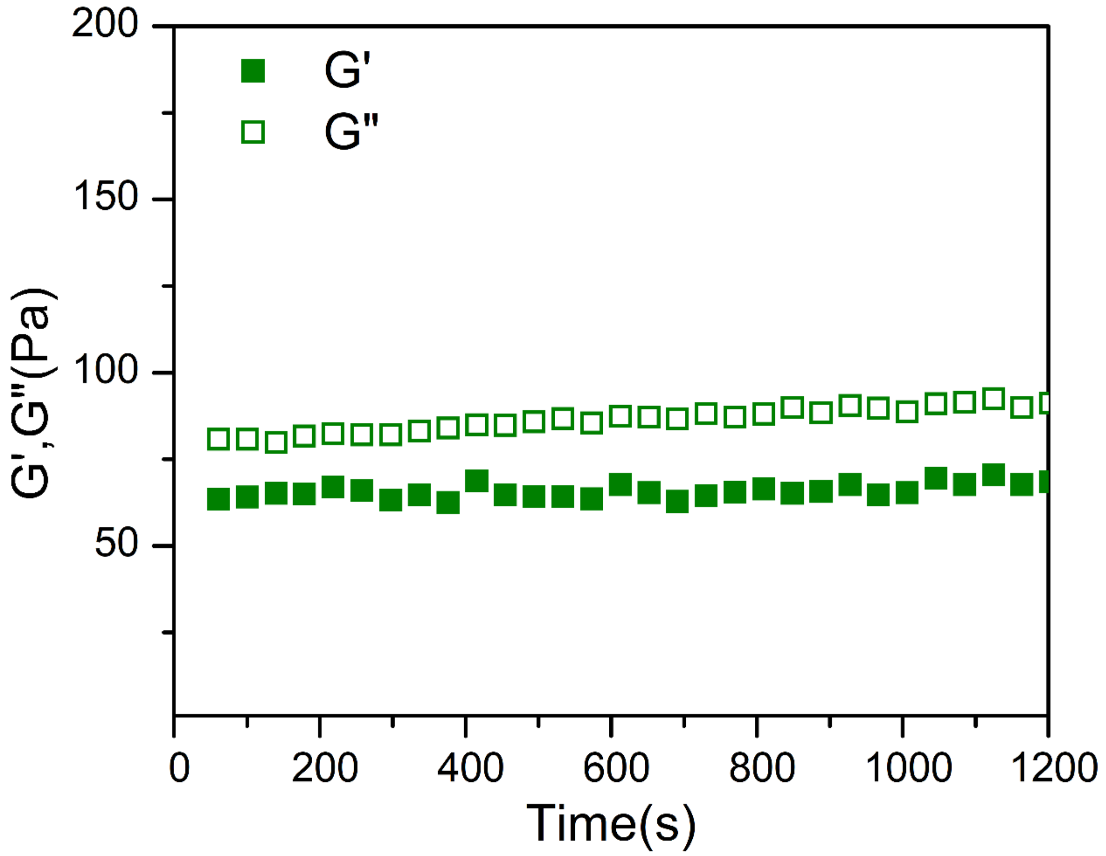

Figure. S7 G' and G" values of **CPAM**/poloxamer composite system.

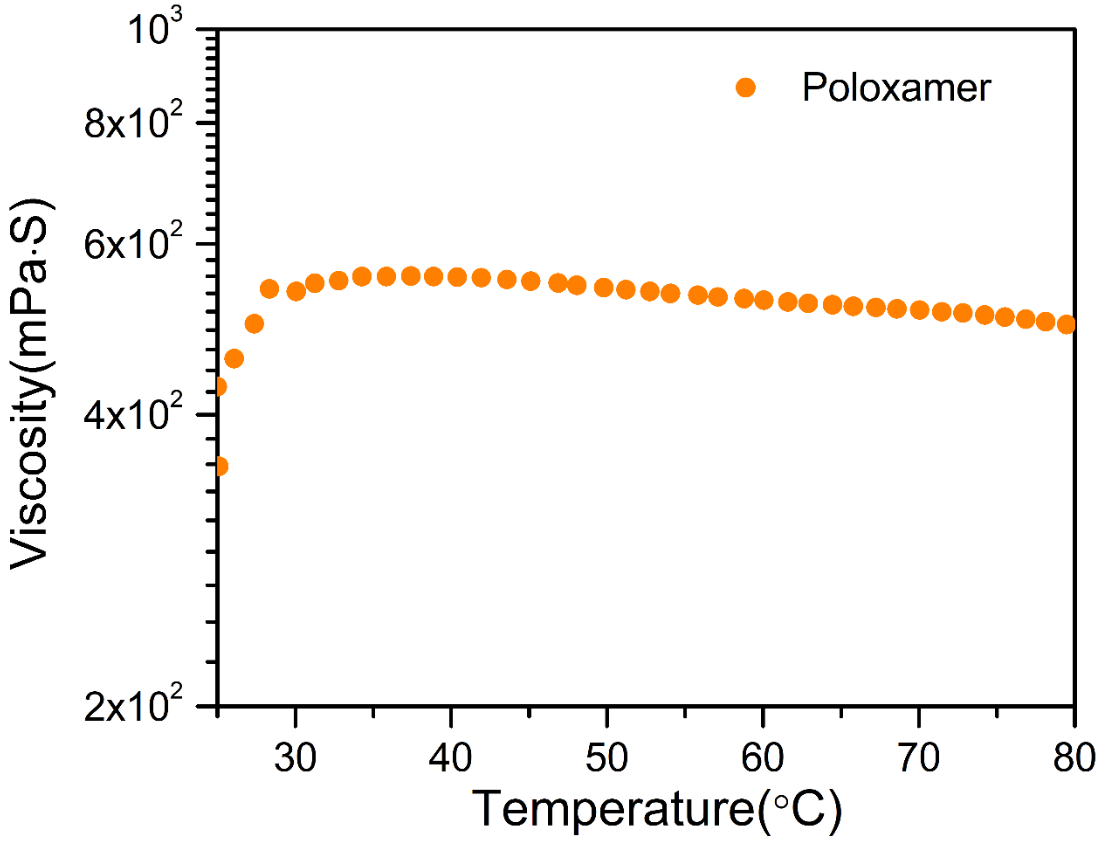

Figure. S8 Viscosity of poloxamer (mass fraction: 10 wt%) in the temperature range of 25-80°C.

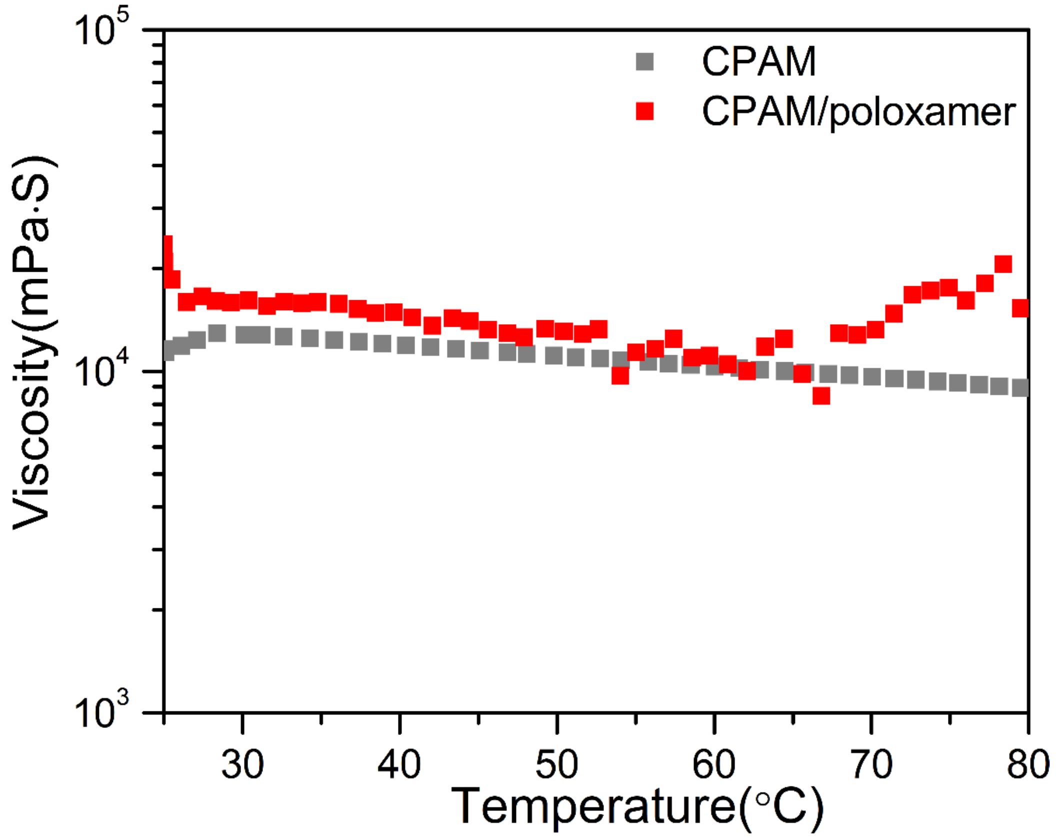

Figure. S9 Viscosity of **CPAM** and **CPAM**/poloxamer.

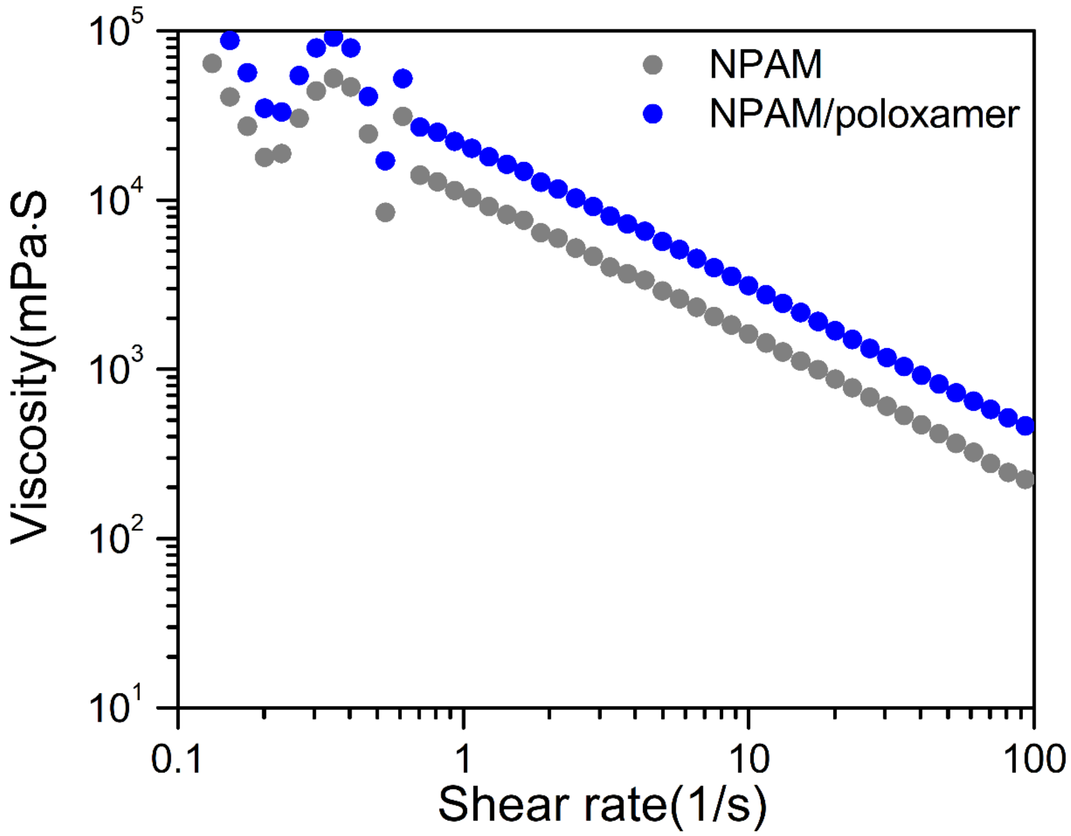

Figure. S10 Viscosity of **NPAM** and **NPAM**/poloxamer.

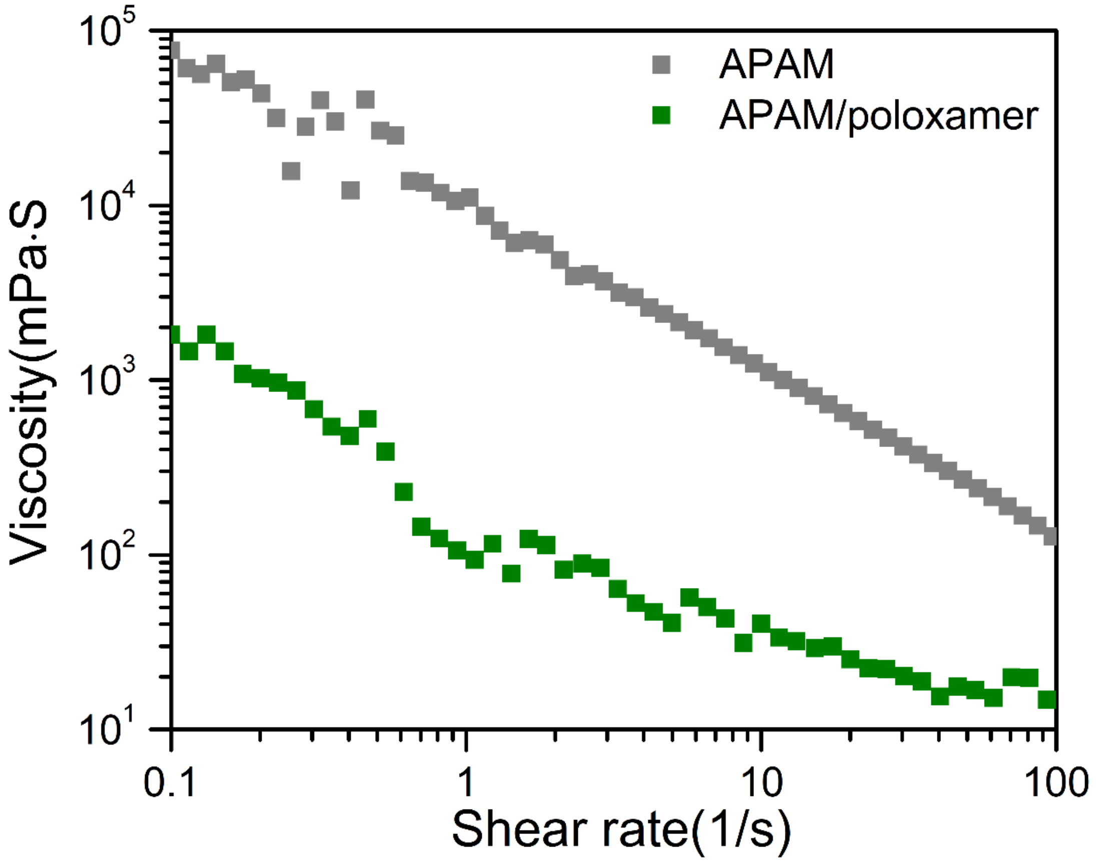

Figure. S11 Viscosity of **APAM** and **APAM**/poloxamer.

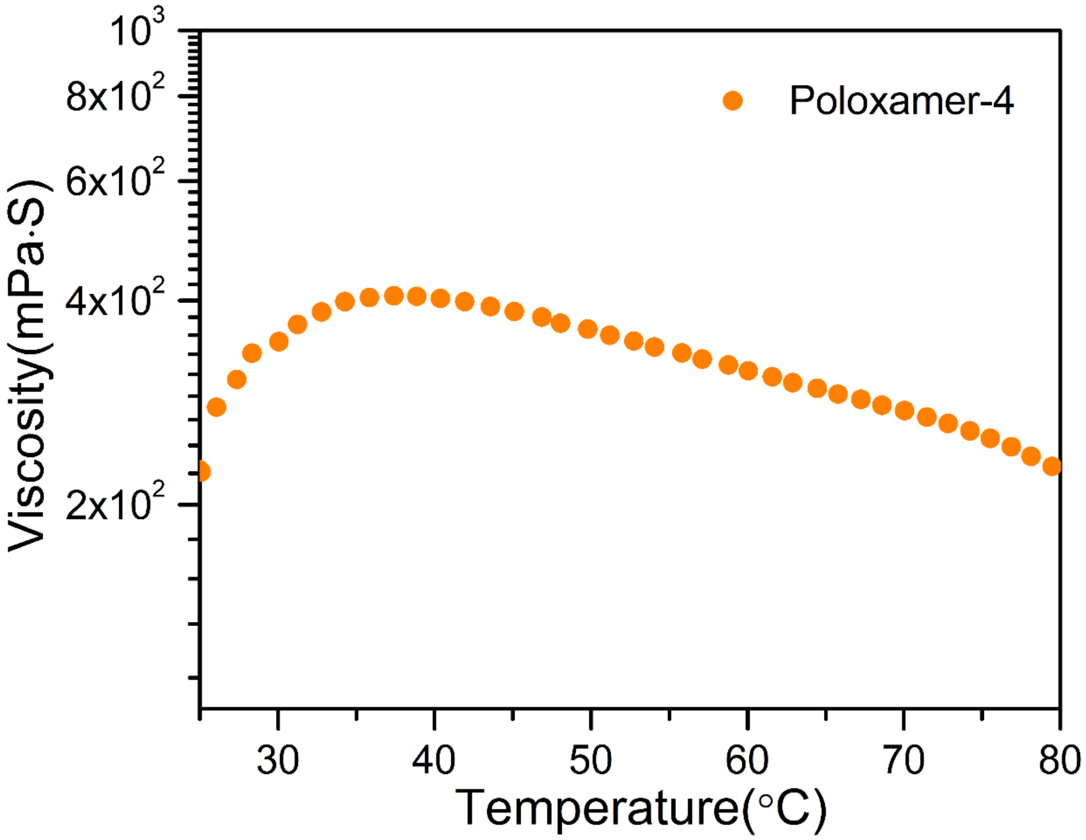

Figure. S12 Viscosity of poloxamer (mass fraction: 4 wt%) in the temperature range of 25-80°C.
